# A Mechanistic Model of NMDA and AMPA Receptor-Mediated Synaptic Transmission in Individual Hippocampal CA3-CA1 Synapses: A Computational Multiscale Approach

**DOI:** 10.3390/ijms22041536

**Published:** 2021-02-03

**Authors:** Pietro Micheli, Rui Ribeiro, Alejandro Giorgetti

**Affiliations:** 1 Department of Biotechnology, University of Verona, 37134 Verona, Italy; pietro.micheli@studenti.univr.it; 2 Institute for Neuroscience and Medicine (INM-9) and Institute for Advanced Simulations (IAS-5), “Computational Biomedicine”, Forschungszentrum Jülich, 52428 Jülich, Germany

**Keywords:** CA3-CA1 synapses, NMDA, AMPA, systems biology, multiscale modeling, Schaffer collateral-CA1 synapses

## Abstract

Inside hippocampal circuits, neuroplasticity events that individual cells may undergo during synaptic transmissions occur in the form of Long-Term Potentiation (LTP) and Long-Term Depression (LTD). The high density of NMDA receptors expressed on the surface of the dendritic CA1 spines confers to hippocampal CA3-CA1 synapses the ability to easily undergo NMDA-mediated LTP and LTD, which is essential for some forms of explicit learning in mammals. Providing a comprehensive kinetic model that can be used for running computer simulations of the synaptic transmission process is currently a major challenge. Here, we propose a compartmentalized kinetic model for CA3-CA1 synaptic transmission. Our major goal was to tune our model in order to predict the functional impact caused by disease associated variants of NMDA receptors related to severe cognitive impairment. Indeed, for variants Glu413Gly and Cys461Phe, our model predicts negative shifts in the glutamate affinity and changes in the kinetic behavior, consistent with experimental data. These results point to the predictive power of this multiscale viewpoint, which aims to integrate the quantitative kinetic description of large interaction networks typical of system biology approaches with a focus on the quality of a few, key, molecular interactions typical of structural biology ones.

## 1. Introduction

Ionotropic glutamatergic receptors are a class of membrane receptors divided into three main subtypes, classified according to their activation to the selective agonists: NMDA (N-Methyl-D-aspartic acid), AMPA (α-amino-3-hydroxy-5-methyl-4-isoxazolepropionic acid), and Kainato. They play a key role in the process of synaptic transmission, which takes place in excitatory glutamatergic synapses, and dysregulations in their normal activities have been widely linked to numerous neurological disorders and synaptopathies [1,2,3,4,5]. Particularly, NMDA and AMPA receptors have been identified as crucial in the molecular mechanism underlying the process of synaptic plasticity, a process that leads to the modulation in the strength of the neuronal response to stimulation, linked to learning and memory [6,7,8].

Complex cognitive functions such as learning and multiple forms of memory are carried out by the hippocampal formation, which can dynamically sample, encode, store, and retrieve information coming from the sensory experience [9,10,11]. The constant encoding and integration of new information is possible thanks to the ability of a neural circuit to continuously reshape its topology and modulate the strength of its connections. In the hippocampal circuits, synaptic plasticity events that individual cells may undergo during synaptic transmissions occur in the form of Long Term Potentiation (LTP) and Long Term Depression (LTD). The trisynaptic circuit, particularly, has been extensively studied because of its apparently simple connectivity and the experimental accessibility of its structures. Inside this pathway, CA3 Shaffer collateral axons innervate CA1 pyramidal cells, forming excitatory glutamatergic synapses. The high density of NMDA receptors expressed on the surface of the dendritic CA1 spines confers to this synapse the ability to easily undergo NMDA receptor-mediated LTP and LTD, which has been substantially evidenced to be essential for some forms of explicit learning in mammals [12,13].

In Schaffer collateral-CA1 synapses, AMPA and NMDA receptors populate the membrane of the CA1 spine, actively participating in synaptic transmission. AMPA receptors are GluR1-GluR4 containing homo/hetero-tetrameric receptors that mediate fast excitatory neurotransmission in glutamatergic synapses. The early phase of synaptic plasticity events that occur in Schaffer collateral-CA1 synapses are associated with alterations in the number of AMPA receptors expressed on the spine membrane through activation of exocytosis or endocytosis mechanisms, as well as changes in AMPA receptors conductance through phosphorylation modifications [14,15]. Together, these molecular mechanisms lead to fine modulations in the strength of the synaptic transmission. The reactions underlying such modulation are controlled by the transient variations in the Ca^2+^ concentration that occur in the post-synaptic spine, especially, due to the activation of NMDA receptors. NMDA receptors are hetero-tetrameric glutamatergic ionotropic receptors permeable to Na^2+^, K^+^, Ca^2+^, and Mg^2+^ ions [16,17]. The permeability to Mg^2+^ ions gives to NMDA receptors a pronounced voltage-dependent behavior. At resting membrane potentials, external Mg^2+^ ions enter into the receptor’s pore but, unlike the other permeating ions, they bind tightly to the pore, blocking it and impairing further ion permeation [18,19]. One of the most accepted physiological mechanisms needed to efficiently unblock NMDA receptors, thus generating an inward Ca^2+^ flux, is a temporal coincidence between the release of pre-synaptic neurotransmitter and a depolarization of the post-synaptic spine (of sufficient amplitude and duration) elicited by post-synaptic activity. This synchronicity is taken into account in the *Spike Timing Dependent Plasticity (STDP)* paradigm that also includes the post-synaptic dendritic activity expressed in the form of *back-propagating action potentials* (bAPs) [20,21]. The transient post-synaptic Ca^2+^ inward current generated by the activation and unblocking of NMDA receptors critically acts on the kinetic equilibrium of the different calcium-binding proteins involved in LTP/LTD-inducing pathways, such as Ca^2+^/Calmodulin-dependent Kinase II (CaMKII) [22,23,24].

Dysfunctions on LTP/LTD-mediated synaptic plasticity have been associated with many neurological disorders such as epilepsy and Alzheimers, Hughtington, and Parkinson’s diseases [4,25,26,27,28,29,30].

A comprehensive and detailed understanding of the molecular mechanisms underlying synaptic transmission and neuroplasticity is then crucial for the physio-pathological characterization of many cognitive functions. However, even if LTP/LTD-mediated synaptic plasticity has been extensively studied, providing a substantial description of a full integration of the interaction networks underlying the whole synaptic transmission, deeply characterized at the molecular level, is currently a major challenge. This could be the starting point for the identification of new therapeutic strategies, aimed at re-tuning the global behavior of the intricate network of molecular interactions underlying synaptic plasticity, thus restoring its functional integrity.

Systems biology models have been shown to be key in approaching the complexity of this type of interaction networks. These models use a holistic approach to unveil the complexity of the molecular pathways and to catalogue all the biological complexes and the relationships between them [31]. They have evolved from empirical descriptions to fundamental mathematical equations applied by computational methods, allowing us to envision how such systems change over time under different conditions. In this way, one can infer qualitative features of the whole system, such as the downstream consequences of a single altered interaction, and consequently identify, for example, pharmacological targets or even predict the severity of a structural variant of a molecular species.

Here, we present, and render available to the scientific community (see Data Availability section), a mathematical model of the CA3 Schaffer collateral-CA1 transmission. Although other integrated and detailed models of glutamatergic synapses have been proposed recently [32,33], a clinical-oriented application of such models, able to also take into account the molecular characterization of particular disease-associated variants, is lacking. The rationale of our work was to provide a synaptic model that can be easily reproduced, run, and be integrated into larger analytical pipelines, proposing a novel viewpoint on the possible applications of comprehensive and detailed system biology models.

Our model allows us to simulate several features of the CA3-CA1 synaptic transmission process. These include (1) glutamate release inside the synaptic cleft as a result of a pre-synaptic stimulation, (2) bAP in the post-synaptic dendritic spine, (3) kinetic description of the gating mechanism of both NMDA and AMPA receptors, (4) estimation of the excitatory post-synaptic currents (EPSCs) and excitatory post-synaptic potentials (EPSPs), including the explicit calculation of the NMDA-mediated inward Ca^2+^ current, and (5) kinetic descriptions of the Ca^2+^-dependent molecular reactions that take place inside the post-synaptic spine and lead to the activation of CaMKII. Here we report some of the qualitative features observed in the receptors-specific contributions to synaptic transmission, as well as in the timing of pre/post-synaptic stimulation. Finally, we offer a further integration of our systems biology approach with a molecular level modeling of disease associated variants. This approach may pave the way to novel multiscale approaches to be used in the pharmacology or structural systems biology field. Because complex biological systems do not rely on individual metabolic networks, having a fully integrated description of metabolic networks allows us to envision the system as a whole instead of a sum of its parts [34]. It follows that the combination of integrated pathways with molecular detail observations, as the one we are presenting here, may bring to light new therapeutic strategies and bring us closer to the new era of personalized medicine.

## 2. Results and Discussions

This section is divided in two main subsections. In the first part, we present the implementation of the mechanistic model, providing an overview on the structure of the pipeline through the description of the individual modules, implemented to describe different fragments of the system. The second part contains the simulation of the model under different parameter configurations. This allows us to infer some qualitative features of the system, with a particular focus on the timing between pre and post-synaptic stimuli, and finally to assess shifts in the global system behavior given by the introduction of rare variants in the NMDA receptors associated with diseases.

### 2.1. An Integrative, Python-Based Pipeline for Simulating Glutamatergic Synaptic Transmission

**Figure 1:**
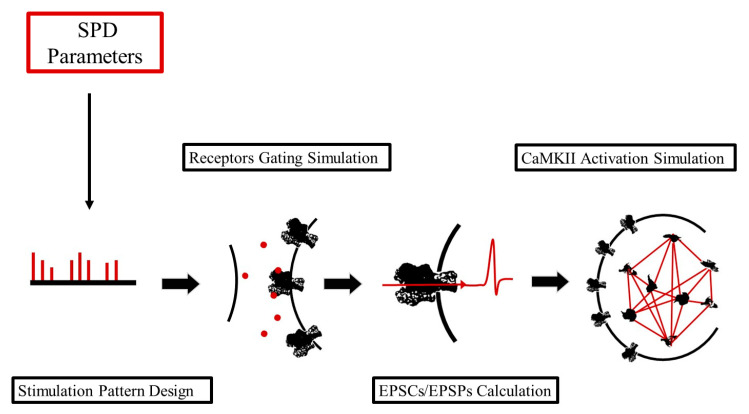
Conceptual scheme of the pipeline to simulate our synaptic transmission model. This scheme illustrates all of the four modules of our framework: (i) Stimulation Pattern Design (SPD module); (ii) Receptors Gating Simulation (RGS module); (iii) Excitatory Post-Synaptic Currents and Potentials (EPSCs/EPSPs) Calculation (CPC module); (iv) Ca^2+^/Calmodulin-dependent Kinase II (CaMKII) Activation Simulation (CAS module).

We developed an integrative mathematical pipeline for the easy running of numerical simulations of synaptic transmission in individual CA3 Schaffer collateral-CA1 synapses, driven by both pre- and post-synaptic stimulation. The pipeline is composed of four different main modules, each one aimed at modelling a different part of the whole transmission process. Starting from the definition of a stimulation pattern, new modules were progressively implemented and added on top of each other, defining a linear pipeline for simulating the synaptic transmission (Figure 1).

#### 2.1.1. Stimulation Pattern Design (SPD)

**Figure 2:**
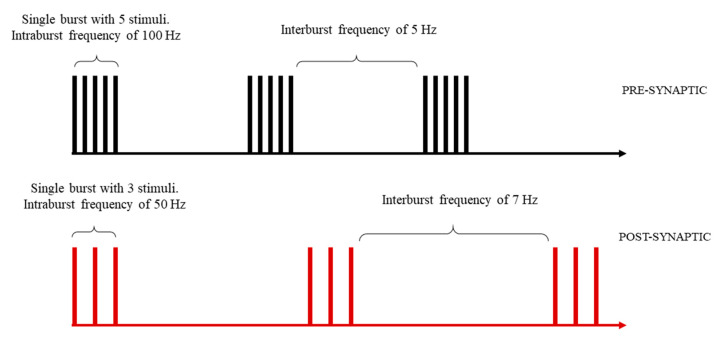
Example scheme of a stimulation pattern. Pre- and post-synaptic stimuli are organized as trains or bursts. Each burst is composed of a sequence of stimuli, delivered at an intra-burst frequency. Inter-burst frequency defines the interval between each burst. Number of stimuli per burst, intra-burst, and inter-burst frequencies can be defined during the stimulation pattern design, for both pre- and post-synaptic patterns.

This module implements a series of functions that easily allow us to define a stimulation pattern that will drive the synaptic transmission. Such stimulation patterns can be composed of both pre- and post-synaptic stimuli, organized as trains of bursts. Here, highly customizable patterns can be designed by setting the number of stimuli composing each burst, the intra-burst, and the inter-burst frequencies for both pre- and post-synaptic stimuli (Figure 2).

Pre-synaptic stimuli are idealized and modeled as the instantaneous rise and fall of the free glutamate concentration in the synaptic cleft, assuming a square pulse-like shape. In this article, we will refer to a pre-synaptic stimulus as a “glutamate pulse”. The quantity of released glutamate (i.e., the pulse amplitude, expressed in μM) and the glutamate exposure time inside the cleft (i.e., the pulse width, expressed in ms) of each pre-synaptic stimulus can be independently parametrized. Post-synaptic stimuli are modeled as dendritic back-propagating action potentials, consisting of transient depolarization potentials of the post-synaptic spine membrane. The shape of such stimuli has been defined using a two-component exponential function (see methods, Section 3.1.1 for further details), as proposed by Shouval et al. [35]. The stimulation pattern defined in this module will constitute the input of the following modules.

#### 2.1.2. Receptors Gating Simulation (RGS)

Pre-synaptic stimuli, defined during the design of the stimulation pattern, are used as input to a second module, which is used to simulate the interactions between the neurotransmitter and the AMPA and NMDA receptors. This module contains the compartmental kinetic description of both the receptor-neurotransmitter binding reactions and the gating mechanisms that lead to the opening of the channels. Particularly, the latter consists of state-transition models (including closed, pre-open, open, and desensitized states) that statistically represent the stochastic distribution of the current traces recorded by electrophysiological experiments. We selected and integrated one kinetic model for both AMPA and NMDA receptors, proposed by Koike et al. and Amico-Ruvio and Popescu [36,37], respectively. Then, we translated both models into systems of first-order differential equations, implemented in a single larger kinetic model using the python PySB package (see methods for further details). Finally, a numerical integration was performed, allowing the simulation of the receptor’s behavior with a high temporal resolution (integration step of 1 μs). We tested the reliability of these *ex-novo* implementations by comparing the behaviors predicted by our model, for both AMPA and NMDA receptors, with the behaviors reported in the works by Koike et al. and by Amico-Ruvio and Popescu [36,37] (Table S1). We observed a strong consistency between the kinetic features of both AMPA and NMDA receptors predicted by our PySB-based model and the respective original models, pointing to a high reliability of our implementation.

#### 2.1.3. EPSCs/EPSPs Calculation (CPC)

The third module of our framework consists of a system of equations used to explicitly calculate the EPSCs and the respective EPSPs generated during the simulation of the synaptic transmission. The EPSCs are estimated by calculating, over the simulation, the ion fluxes that permeate each open channel (predicted with the RGS module described in Section 2.1.2). This estimation is made according to the channel-specific conductance, the channel-specific reversal potential, and the depolarization level of the post-synaptic membrane. The EPSPs are then derived from the EPSCs (see methods Section 3.1.3 for further details). All the depolarization potentials, which include the EPSPs and, eventually, the bAPs arising from the post-synaptic stimulation, are summed together to assess the global changes in the membrane depolarization value. In this module, the equation for the explicit estimation of the NMDA-mediated Ca^2+^ current is used to assess the post-synaptic changes in the Ca^2+^ concentration according to a simple model proposed by Shouval et al. [35] (see methods, Section 3.1.3 for further details).

#### 2.1.4. CaMKII Activation Simulation (CAS)

The last module of our pipeline aims to simulate a kinetic description of the post-synaptic molecular interactions that controls the CaMKII kinase autophosphorylation events. For this purpose, as previously described for the RGS module (Section 2.1.2), we selected from the literature a detailed kinetic model based on its reproducibility, and we transcribed all its reactions into a second PySB model as a system of first-order differential equation. We chose to implement a model for the CaMKII activation proposed by Pepke et al. [38], and we integrated it into the simulation pipeline. This kinetic model includes a large number of reactions, mainly characterizing the interactions between free Ca^2+^ ions, calcium-binding messenger CaM, and the CaMKII enzyme. Particularly, the Ca^2^-CaM mediated autophosphorylation of CaMKII enzyme, which leads to its own activation, directly plays a pivotal role in inducing the early phase of synaptic plasticity [22,23,24]. Although the changes in the synaptic strength are currently not explicitly assessed in our model, the variations in the activated CaMKII accumulation allows one to assess the relative efficiency of the simulated synaptic transmission.

### 2.2. Kinetic Behavior Analysis of AMPA and NMDA Receptors under Different Pre-Synaptic Stimulation Conditions

We explored how AMPA and NMDA receptors kinetically behave under different stimulations patterns, exploiting the RGS module (Section 2.1.2). For this purpose, we simulated the model using different pre-synaptic stimulation patterns, consisting of either a single glutamate pulse or bursts of multiple glutamate pulses, delivered at different frequencies (ranging from 10 to 100 Hz). The amplitude of the glutamate pulses was set into a physiological range of 1–2 mM [39,40], while the time width was varied in a range between 1 ms and 1.5 s.

**Figure 3:**
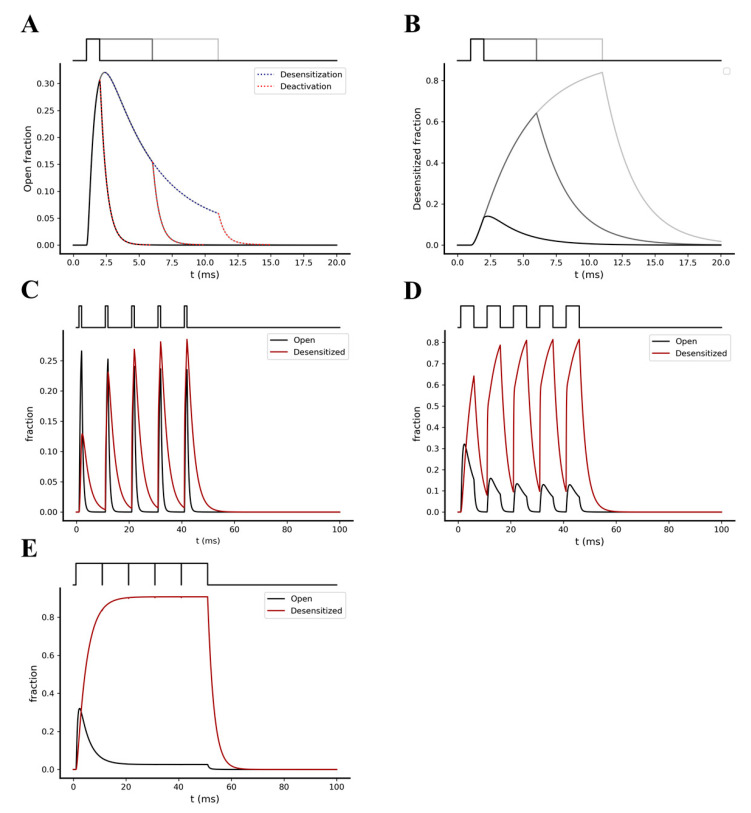
Opening and desensitization kinetics of the AMPA receptors. (**A**) Open fraction kinetics following a stimulation with a single glutamate pulse of 1 mM amplitude and width of 1 ms (black), 5 ms (dark grey) and 10 ms (light grey). Blue dotted trace shows the desensitization kinetics, while red dotted traces show the deactivation kinetics following glutamate removal from the synaptic cleft. (**B**) Desensitized fraction kinetics following a stimulation with a single glutamate pulse of 1 mM amplitude and width of 1 ms (black), 5 ms (dark grey), and 10 ms (light grey). (**C**–**E**) Kinetics of open and desensitized fractions following pre-synaptic stimulations with a burst composed of 5 glutamate pulses, with glutamate pulse amplitude of 1 mM, an intra-burst frequency of 100 Hz, and a pulses width of 1 ms (**C**), 5 ms (**D**), and 10 ms (**E**).

We first focused on the kinetic behavior of AMPA receptors under a single glutamate pulse of 1 mM, simulated with 1, 5, and 10 ms width. The desensitization kinetics of AMPA receptors predicted by the gating model shows a much slower time course (τ = ~25 ms, fitted with single exponential function) compared to the deactivation kinetics (τ = ~0.55 ms, fitted with single exponential function) after the end of a single glutamate pulse (Figure 3A). Moreover, both the exposure time of the glutamate (defined by the pulse width) and the stimulation frequency seem to strongly affect the number of desensitized receptors reached after a single pre-synaptic event [36] (Figure 3B). The faster deactivation, compared to the desensitization predicted by the model, points to the property of AMPA receptors to preferentially undergo a temporal accumulation of desensitized states instead of the open states.

We then analyzed how the variation of the glutamate pulses duration affects the summation of desensitized states under a single pre-synaptic burst stimulation. The latter was simulated by a single burst composed of 5 glutamate pulses of 1 mM amplitude and 1,5, and 10 ms width, with an intra-burst frequency of 100 Hz. We observed a significant increase in the temporal summation of desensitized AMPA receptors as the glutamate exposure values increased (Figure 3C–E, respectively).

**Figure 4:**
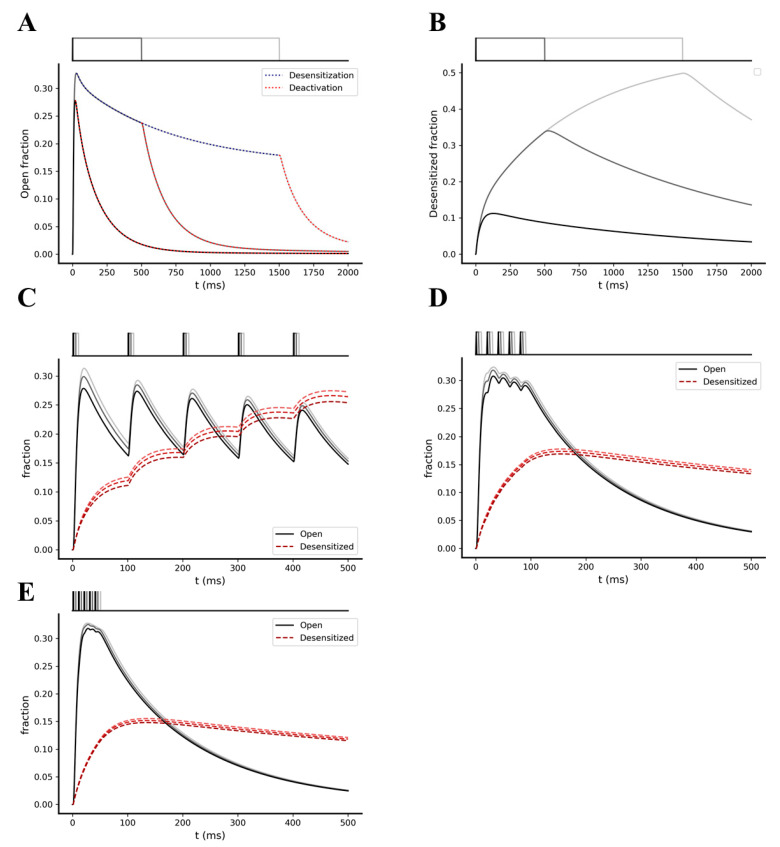
Opening and desensitization kinetics of the NMDA receptors. (**A**) Open fraction kinetics following a stimulation with a single glutamate pulse of 1mM amplitude and width of 1 ms (black), 500 ms (dark grey), and 1.5 s (light grey). Blue dotted trace shows the desensitization kinetics, while red dotted traces show the deactivation kinetics following glutamate removal from the virtual synaptic cleft. (**B**) Desensitized fraction kinetics following a stimulation with a single glutamate pulse of 1mM amplitude and width of 1 ms (light grey), 5 ms (dark grey), and 10 ms (black). (**C**–**E**) Kinetics of open and desensitized fractions following pre-synaptic stimulations with a burst composed of 5 glutamate pulses, with glutamate pulse amplitude of 1 mM, pulse width of 1 ms (black), 5 ms (dark grey), and 10 ms (light grey) and an intra-burst frequency of 10 Hz (**C**), 50 Hz (**D**), and 100 Hz (**E**).

Next, we analyzed the predicted kinetic behavior of NMDA receptors. By simulating a single glutamate pulse of 1 mM amplitude and 1 ms, 500 ms, and 1.5 s width, we observed a significatively slower deactivation and desensitization kinetics compared to AMPA receptors (Figure 4). Fitting the curves with a single exponential function, we found time constants of 163, 195, and 210 ms for the deactivation kinetics after 1 ms, 500 ms, and 1.5 s of glutamate exposure, respectively, and a time constant of 1.95 s for the desensitization kinetics (Figure 4A,B). From these results, we got a ratio between the desensitization and the deactivation time constant (τ_desens_/τ_deact_) of ~12 for the NMDA receptors and ~45 for the AMPA receptors. The lower value found for the NMDA receptors leads to a more efficient temporal summation of its open states. In fact, when we simulated the model with a single pre-synaptic burst of 5 glutamate pulses of 1 mM amplitude and 1, 5, and 10 ms width, with intra-burst frequencies of 10, 50, and 100 Hz, we observed, effectively, summation of the open NMDAs (Figure 4C–E).

**Figure 5:**
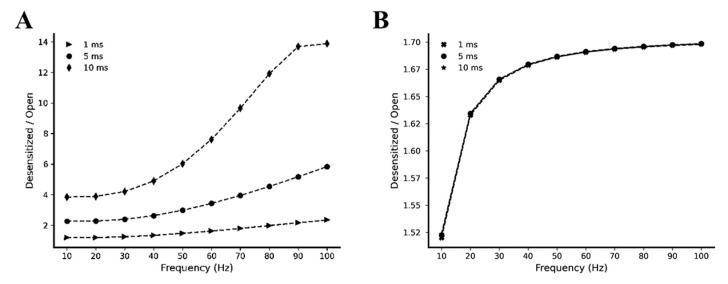
Desensitized/Open ratio expressed as a function of stimulation frequency. Simulations were performed using single pre-synaptic glutamate pulses of 1 mM amplitude and 1, 5, and 10 ms width. For each simulation, the ratio between the desensitized and the open fraction has been calculated for (**A**) AMPA (triangles, circles and diamonds symbols refer, respectively, to 1, 5 and 10 ms pulse width) and (**B**) NMDA receptors (crosses, circles and stars symbols refer, respectively, to 1, 5 and 10 ms pulse width).

To have a better insight into the difference between the kinetic behavior of AMPA and NMDA receptors, we simulated our model with a single pre-synaptic burst of 5 glutamate pulses of 1 mM amplitude and 1, 5, and 10 ms width, varying the intra-burst frequency between 10 and 100 Hz. For each intra-burst frequency, we calculated the ratio between the total number of desensitized and open receptors. According to our model, these simulations pointed out that the desensitized/open ratio of AMPA receptors depends more on the stimulation frequencies and on the glutamate pulses durations compared to the desensitized/open ratio of the NMDA receptors (Figure 5).

### 2.3. Temporal Relationship between Pre- and Post-Synaptic Stimuli Strongly Impacts Synaptic Transmission Efficiency

During the stimulation of the synapse, the equations implemented in the CPC module (Section 2.1.3) allow us to explicitly assess the individual contribution of both AMPA and NMDA receptors to the global electrical transmission. Pre-synaptic-induced excitatory potentials and post-synaptic dendritic back-propagation events, programed during the stimulation pattern design, are integrated together to continuously estimate the variations in the NMDA permeability, as well as in the Ca^2+^ flux driving force (see methods Section 3.1.3 for further details). We explored, through several simulations, how the temporal relationship between pre- and post-synaptic stimuli can shape the efficiency of the electro-chemical transmission.

#### 2.3.1. AMPA-Mediated EPSPs Are Not Sufficient to Efficiently Relieve the Mg^2+^ Block from NMDA Receptors

The pronounced voltage-dependent affinity of NMDA receptor for the extracellular Mg^2+^ ions causes the actual permeation of the channel to be strongly modulated by the depolarization level of the membrane [19]. We have previously observed that the kinetic equations implemented in the RGS module predict no effective temporal summations of open AMPA receptors because of their fast desensitization and deactivation kinetics, as observed in other studies [36,41]. Analyzing the output of the RGS module using the equations implemented in the CPC module (Section 2.1.2 and Section 2.1.3), we then observed that, coherently, the AMPA-mediated responses also tend not to summate (Figure S1).

**Figure 6:**
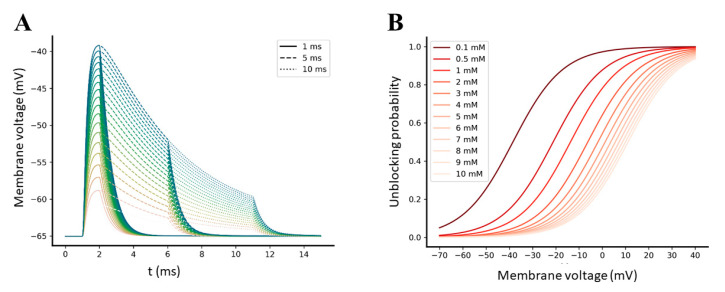
(**A**) Simulated AMPA-mediated EPSPs evoked by different numbers of available AMPA receptors, ranging from 20 (lower trace) to 200 (upper trace). Solid, dashed, and dotted traces refer to single pulse stimulation performed with a glutamate pulse width of, respectively, 1, 5, and 10 ms. (**B**) Sigmoidal unblocking probability function for Mg^2+^ block, expressed as a function of membrane voltage. Each trace corresponds to a different value of extracellular Mg^2+^ concentration.

This observation prompted us to investigate if the amplitude of an AMPA-mediated EPSP evoked by a single pre-synaptic event was high enough to relieve the Mg^2+^ block from NMDA receptors. Because the EPSPs amplitudes of AMPA and NMDA receptors are influenced by their levels of expression on the post-synaptic spine surface, we performed multiple simulations of a single glutamate pulse of 1 mM amplitude and 1, 5, and 10 ms width, varying the level of available AMPA receptors in a range between 20 and 200 [42]. Simulation results reported that the maximum AMPA-mediated EPSPs peaks elicited by single-pulse pre-synaptic stimulations reach −40 mV with 200 units of AMPA receptors (Figure 6A). According to the Mg^2+^ unblocking probability function that we have incorporated into the model (see methods Section 3.1.3 for further details), such a depolarization level can effectively release the Mg^2+^ ion from NMDA receptors only if the extracellular Mg^2+^ concentration is very low compared to the physiological concentration (Figure 6B), which is near to 1 mM [19].

These results emphasize the fact that pre-synaptic events on their own may not be enough to ensure an effective Ca^2+^ permeation. As supported by the STDP paradigm, temporal coordination between pre- and post-synaptic events must occur in order to allow a significant Ca^2+^ influx that can effectively trigger plasticity [43].

#### 2.3.2. Synchronization between Pre- and Post-Synaptic Stimulation Significantly Increases the NMDA Receptor Contribution to Synaptic Transmission

**Figure 7:**
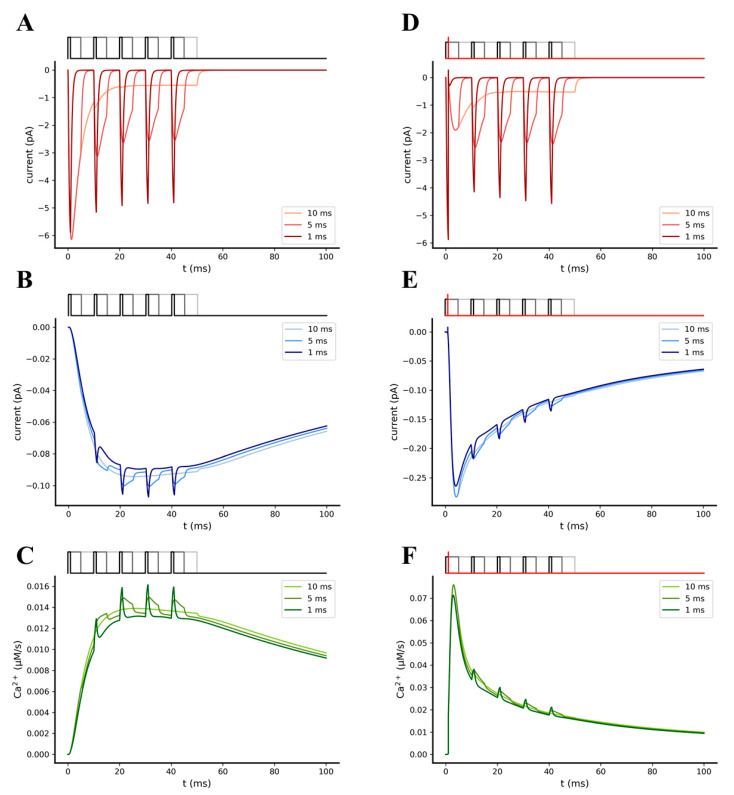
Simulation of synaptic transmission elicited by a single pre-synaptic burst of 5 glutamate pulses, in the absence of (**A**–**C**) or in the presence of (**D**–**F**) a single post-synaptic back-propagating action potential (bAP). (**A**,**D**) Time course of the individual AMPA-mediated EPSC. (**B**,**E**) Time course of the individual NMDA-mediated EPSC. (**C**,**F**) Time course of the Ca^2+^ molar flowrate that permeate NMDA receptors during the simulations. Pre-synaptic bursts were composed of 5 glutamate pulses of 1 mM amplitude and 1 ms (black pulses), 5 ms (dark grey pulses), and 10 ms (light grey pulses) width; in each plot, the responses elicited by 1 to 10 ms widths are represented by different colors, respectively, from the darkest to the brightest. Post-synaptic activity (red trace) was programmed as a single dendritic back-propagation event that occurs 1 ms after the first pulse of the pre-synaptic burst began. Both simulations were performed in the presence of 20 AMPA, 15 NMDA, and 1 mM of extracellular Mg^2+^.

We further investigated how the synchrony between pre- and post-synaptic activity can affect the efficiency of synaptic transmission, particularly by increasing the amplitude of the NMDA receptors-mediated EPSCs and EPSPs. For this purpose, we compared the individual responses of the AMPA and NMDA receptors obtained from two different stimulation patterns, one including only pre-synaptic stimulation and one including coupled pre- and post-synaptic stimulations. In both stimulation pattern, the pre-synaptic stimulation consisted of a single theta burst composed of 5 glutamate pulses of 1 mM amplitude and 1, 5, and 10 ms width, with an intra-burst frequency of 100 Hz [44]; post-synaptic stimulation was designed as a single dendritic back-propagation event, which occurs in the post-synaptic spine 1 ms after the first pre-synaptic stimuli was delivered. Simulations were performed in the presence of 20 AMPA and 15 NMDA receptors [42,45], with extracellular Mg^2+^ concentration set to 1 mM. As expected, significant increases in the total NMDA receptor-mediated current peak (~2.5 fold), as well as in the Ca^2+^ that permeated the channel (~4.5 fold), were observed during the coupled pre- and post-synaptic stimulation compared to the pre-synaptic stimulation alone, showing the impact of bAP-mediated synaptic facilitation on the NMDA receptors conductance (Figure 7).

**Figure 8:**
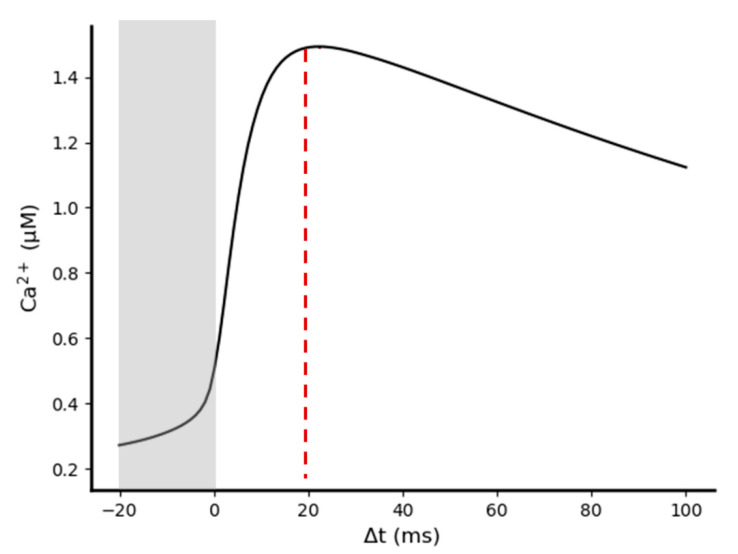
Relationship between pre/post-synaptic stimulation timing and the Ca^2+^ concentration peaks reached in the post-synaptic spine. Simulations were performed in the presence of 20 AMPA, 15 NMDA, and 1 mM of extracellular Mg^2+^. Maximum post-synaptic Ca^2+^ concentration was reached with Δt ≈ 20 ms. Gray rectangle highlights negative Δt values in which post-synaptic stimuli precede pre-synaptic stimuli.

Because we had observed that the presence of a bAP during stimulation significantly increases the NMDA receptor mediated EPSC, we analyzed how variations in temporal coordination level between pre- and post-synaptic stimuli impacts the amplitude of the elicited Ca^2+^ influx. For this purpose, we performed multiple simulations varying the time interval between pre- and post-synaptic stimuli (Δt = t_post_ − t_pre_). For each simulation, we then evaluated the effect of the bAP-induced synaptic facilitation by calculating the maximum Ca^2+^ concentration reached in the post-synaptic spine. Simulating a single pre-synaptic glutamate pulse of 1 mM amplitude and 1 ms width, together with a single post-synaptic bAP, we found that post-synaptic Ca^2+^ rises from a value of ~200 nM (the post-synaptic Ca^2+^ concentration elicited by a single pre-synaptic event alone) to a maximum of ~1.4 μM (Figure 8). This value is obtained when the pre-synaptic event precedes the post-synaptic event (positive Δt) of ~20 ms, in agreement with the Hebbian STDP paradigm for synaptic plasticity (see *Feldman 2012* [20] for a review).

### 2.4. Kinetic and Pharmacological Analysis of NMDA Variants: Multiscale Integration

Deactivation time course defines the time required by the receptor-mediated current to decay after the removal of the agonist from the synaptic cleft. This kinetic feature, together with EC_50_ values of the agonist, constitute a prominent quantitative feature used to perform functional analysis of ion channels [46]. Many published studies on rare NMDA receptor variants have tried to assess the severity of a certain mutation, considering its impact on both glutamate potency and deactivation time constant [25,47,48,49].

**Figure 9:**
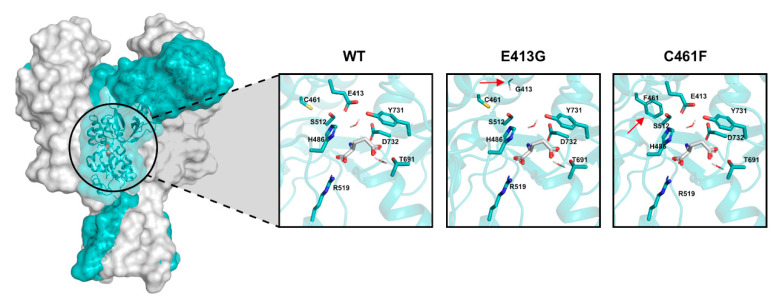
Structure of human GluN1/GluN2A NMDA receptor (PDB accession code: 4TLM). The GluN2B subunit is colored in light blue. The insights show the glutamate binding domain of the wild-type (WT) receptor and the two structural variants Glu413Gly (E413G) and Cys461Phe (C461F). Each window focuses on the docked glutamate (white molecule) and the crucial residues that directly participate to the interaction. Red arrows point to the residue substitution of each of the two structural variants.

We used our model to predict the glutamate affinity (K_d_) and the weighted deactivation time constant (τ_w_) in NMDA receptor variants, based on the EC_50_ values that have been reported in different experimental and computational studies [25,47,48,50]. In particular, we focused on two rare variants: Glu413Gly and Cys461Phe that fall inside the GluN2B binding pocket (Figure 9). These variants have been shown to decrease the glutamate potency, which may result from a decrease in the glutamate affinity [47,48,50,51].

Therefore, we tuned the NMDA kinetic model to reproduce the same concentration-response behaviors experimentally observed for both the Glu413Gly and Cys461Phe variants.

Exploiting our kinetic model, we were able to computationally assess the NMDA-glutamate concentration-response relationship by using the following approach: firstly, we sampled concentration values in a range between 0.01 and 1000 mM; next, for each value, we ran the RGS module, simulating a single glutamate pulse, with amplitude corresponding to the current glutamate concentration value and width of 1.5 s, as reported by experiments [47], setting the number of AMPA receptors to 0 (because we were interested in isolating the NMDA response). Finally, calculating from each simulation the peak of the evoked current, EC_50_ value was obtained by fitting the concentration-response data with the logistic function.

To predict the shifts in the NMDA receptor-glutamate affinity associated with the rare variants Glu413Gly and Cys461Phe, knowing their experimental EC_50_ values (75–79 μM for Glu413Gly [47,48] and 169 μM for Cys461Phe [47]), we progressively increase, during a sequence of multiple simulations, the ratio between the rate constants k_off_ and k_on_ (i.e., the K_d_) of the equations describing the interaction between the NMDA receptor and the glutamate. For each simulation, we computed the EC_50_ value, and at the end of all the simulations we selected the K_d_ that rendered the EC_50_ values closest to the experimental ones.

As a result, we found that the NMDA receptor kinetic behavior generated by predicted K_d_ values shows a current deactivation time constantly very close to the experimental ones (Table 1).

The kinetic model of the NMDA receptor was tuned by only increasing the k_off_ rate constant of the glutamate binding reactions. Therefore, we reasoned that the coherence between our results and the experimental data points to the fact that the analyzed variants are likely to affect the affinity of the receptor (thus causing an EC_50_ shifting) by negatively altering the glutamate residence time inside the binding pocket of the receptor.

**Figure 10:**
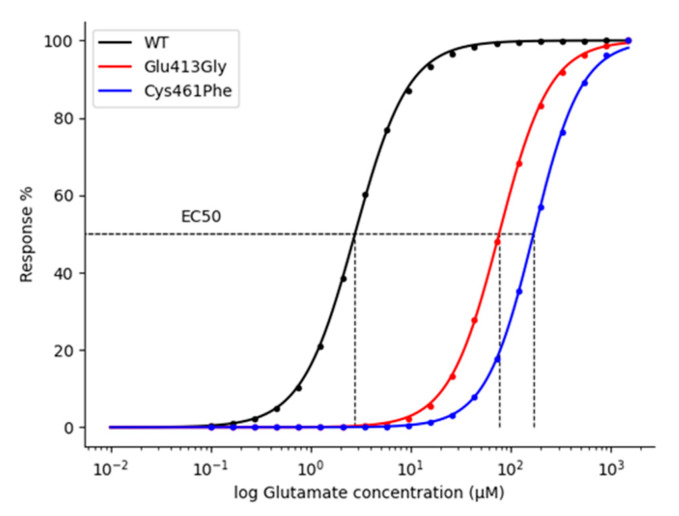
Dose-response curves of the effect of glutamate on wild-type (WT) and variant-NMDA receptors. Simulation data was fitted with logistic regression. EC_50_ values of 2.7, 76, and 169 μM for WT, Glu413Gly, and Cys461Phe-NMDA receptors, respectively.

For the wild-type NMDA receptor, we found a K_d_ value of 2.5 μM and a deactivation constant of 328 ms, whereas, for the Glu413Gly and Cys461Phe variants, we found K_d_ values of 190.5 and 446.5 μM and deactivation constants of 29 and 27 ms, respectively (Figure 10 and Table 1). As these results imply, the Glu413Gly and Cys461Phe variants increase the K_d_ of the glutamate ~75 and ~180-fold (Table 1).

**Figure 11:**
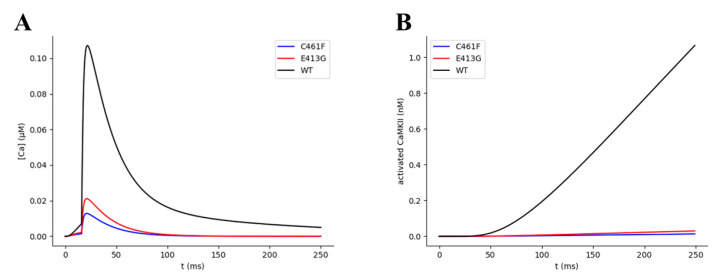
Variation of (**A**) Ca2+ concentration and (**B**) activated CaMKII over time for the WT and variant NMDA receptors. All simulations were performed under one pair of single pre- and post-synaptic pulses, with a pre-synaptic pulse of 1 ms of glutamate exposure, a delay between the pre- and the post-synaptic stimuli of 20 ms, and 1 mM of Mg^2+^.

The next step in our multiscale analysis of NMDA Glu413Gly and Cys461Phe receptor variants consisted in further investigating if the calculated affinity alterations can impact the synaptic plasticity mechanism. To address this question, we simulated the effects of the structural variants on the amplitude of the rise in the post-synaptic Ca^2+^ concentration and on the amount of activated CaMKII, an enzyme that directly plays a pivotal role in triggering synaptic plasticity events in CA3-CA1 synapses. This latter estimation was done by exploiting the CAS module (Section 2.1.4). This module contains a mathematical description of the Ca^2+^-dependent CaM-CaMKII transduction pathway, which, starting from Ca^2+^ transients, leads to activation of CaMKII kinase (see methods Section 3.1.4 for further details). We stimulated our virtual synapse with a pair of single pre- and post-synaptic stimuli (glutamate pulse of 1 mM amplitude and 1ms width, time interval between pre- and post-synaptic stimuli of 20 ms). As expected, we found that the predicted decrease in the NMDA glutamate affinity significatively attenuates the amplitude of the elicited post-synaptic Ca^2+^ variation ~5 and ~8.5 fold for the Glu413Gly and Cys461Phe variants, respectively (Figure 11A). Moreover, the kinetic model for the Ca^2+^-mediated activation of the CaMKII enzyme predicted much lower amounts of activated CaMKII for Glu413Gly (~13 fold) and Cys461Phe (~23 fold) variants compared to the wild type (Figure 11B). Considering the key role that the CaMKII enzyme plays in the molecular mechanism underlying the synaptic plasticity process, the predicted drastic decrease in the activation efficiency of such enzyme points out the severity of these rare structural variants. In fact, because CaMKII-driven neuroplasticity seems to be negatively affected in a significant way, severe neuropathological phenotypes, including learning and memory impairment, are likely to arise.

**Figure 12:**
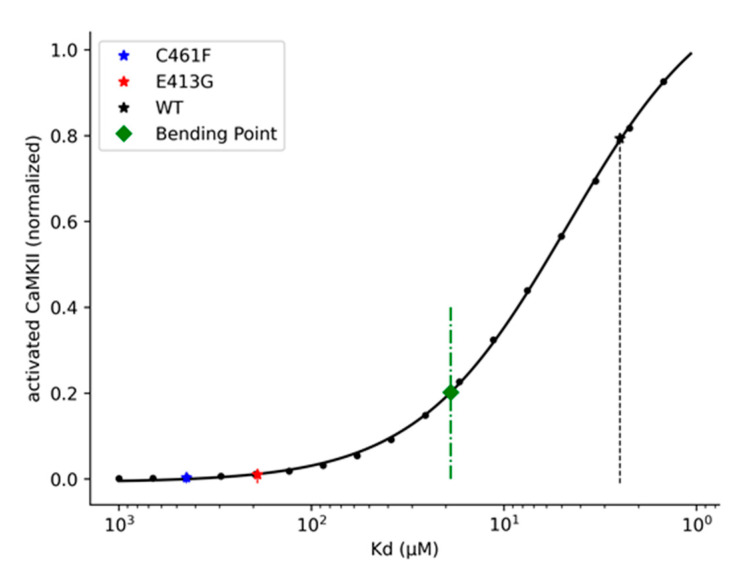
Variation of activated CaMKII as a function of the NMDA-glutamate K_d_ values. All simulations were performed under one pair of single pre- and post-synaptic pulses, with a pre-synaptic pulse of 1 ms of glutamate exposure, a delay between the pre- and the post-synaptic stimuli of 20 ms, and 1 mM of Mg^2+^.

In the last part of our in silico experiment, we were interested in reporting a more general representation of the relationship between NMDA-glutamate affinity and CaMKII enzyme activation efficiency. Here, our rationale was to search for an NMDA-glutamate affinity threshold that can be used for discriminating between high and low-severity variants, knowing their respective K_d_. We proceeded, for this purpose, to simulate the whole synaptic model with the same basic stimulation pattern previously adopted for the analysis of the Glu413Gly and Cys461Phe variants but varying the K_d_ affinity value in a range between 1 and 1000 μM. For each simulation (i.e., for each K_d_ value), we selected the maximum amount of activated CaMKII observed. Data were first normalized to the maximum response observed across all the simulations and then fitted with the four-parameter logistic function (see methods Section 3.2.3 for further details) (Figure 12). Finally, the threshold was calculated by finding the bending point of the fitted curve, which corresponded to a K_d_ value of ~19 μM (Figure 12).

The identification of this type of thresholds can be very useful for a rapid assessment of the downstream effects of variants and can be easily integrated into larger analytical pipelines. We are currently working on a further implementation of this synaptic model that also integrates a detailed kinetic description of the reactions controlling the phosphorylation of AMPA receptors by the CaMKII enzyme, an event which is known to directly control synaptic strength modulations (LTP and LTD) by altering the conductance and trafficking of these receptors. With this further extension, we aim to explicitly quantify synaptic plasticity events that can occur during the stimulations.

## 3. Methods

In this section, we provide a full and detailed description of all the individual modules that compose the proposed mathematical model, each of which implements a different fragment of the whole synaptic transmission process.

This modular rationale at the base of the framework implementation guarantees an easy customization of the simulation pipeline, as well as the further extensibility of the system.

The current build of the framework includes:

- Stimulation Pattern Design (SPD) module, where both pre- and post-synaptic stimuli can be programmed independently. This module allows us to define the inputs of the virtual synapse.
- Receptors Gating Simulation (RGS) module. This module performs a compartmentalized kinetic simulation of the virtual synaptic cleft, where a neurotransmitter released from pre-synaptic stimuli interacts with ionotropic membrane receptors expressed on the post-synaptic spine.
- EPSCs/EPSPs Calculation (CPC) module. This module analyzes the data coming from the RGS module and, calculating synaptic currents and their respective potentials, integrates pre- and post-synaptic stimuli. It constitutes a “bridge” between the extracellular and the intracellular compartments.
- CaM/CaMKII Activation Simulation (CAS) module. This module performs a compartmentalized kinetic simulation of a set of molecular reactions that takes place in the virtual post-synaptic spine, which includes the interactions between Ca^2+^, Calmodulin (CaM), and Ca^2+^/CaM-dependent Kinase II (CaMKII).

The kinetic equations used to describe the reactions contained in both the RGS and CCS modules are implemented, exploiting the PySB python package [53] as systems of first-order differential equations. Numerical integration is performed using the SciPy ODE integrator [54]. All of the data analysis and fittings were performed using SciPy and Numpy packages [54,55]. Finally, all the plots were generated using the Matplotlib library [56].

All the code is stored in a publicly available github repository (https://github.com/pietromicheli/CA3-CA1_SynapticModel), where a jupyter notebook file for running simulations and performing basic analysis can also be found.

### 3.1. Mathematical Model Implementation

#### 3.1.1. SPD Module

In this module, the stimulation pattern of the virtual synapse can be designed. Bidirectionality is a crucial feature of neuronal communication. The functional and topological properties of the brain neural network can be significantly shaped by the temporal relationship between forward and backward signals, as the STDP paradigm for the synaptic plasticity claims [20,21,57]. Therefore, integration of pre- and post-synaptic stimuli constitute a logic core of our implementation. For this purpose, patterns of pre- and post-synaptic stimuli can be programmed and simulated independently in order to analyze how the system behaves under different levels of synchronization between pre- and post-synaptic activities. Each pattern is modeled as a train of bursts. Numbers of stimuli per burst, intra-burst, and inter-bursts frequencies can be specified to design custom stimulation patterns (Figure 1).

In our model, pre-synaptic stimuli have been idealized as glutamate pulses, representing the instantaneous rise and fall in the free neurotransmitter concentration available inside the cleft compartment following pre-synaptic action potentials. Amplitude (i.e., the amount of available free glutamate) and width (i.e., the exposure time of the free glutamate) of the pre-synaptic glutamate pulses can be defined during the stimulation design.

On the other hand, post-synaptic stimuli have been modeled as transient depolarizations of the post-synaptic spine generated by dendritic back-propagating action potentials (bAP). Each bAP is shaped using a two-component exponential function, taken from the work by Shouval et al. [35]:

(1) $$\mathrm{bAP}\left(t\right)=V_{max}*\left(\left(I_{fast}*exp\left(\frac{-t}{\tau_{fast}}\right)\right)+\left(I_{slow}*exp\left(\frac{-t}{\tau_{fast}}\right)\right)\right)$$

where *V_max_* is the maximum depolarization value for bAP value set to +67 mV [58], *I_fast_* and *I_slow_* are the relative magnitudes of the fast and slow components of the bAP that sum to one, and τ*_fast_* and τ*_slow_* are the relative time constants that describe the exponential decays of the two components.

#### 3.1.2. RGS Module

This module contains a system of kinetic equations describing the interactions between glutamate and AMPA/NMDA receptors, which take place inside the cleft compartment. The aim of this module is to accurately simulate both the receptors-neurotransmitter binding reactions and the gating mechanism that lead to opening or desensitization of the receptors.

Individual models describing the kinetic behavior of both AMPA and NMDA receptors have been selected from the literature based on their reproducibility and subsequently implemented as systems of first-order differential equations inside the PySB framework. To reproduce the kinetic behavior of AMPA receptors, we chose a model proposed by Koike et al. [36] for homomeric GluR2(flip) receptors. The model assumes two glutamate binding steps, one pre-open transient state, three desensitized states, and one open state of the receptor (Figure S2B). For the kinetic description of the gating mechanism of NMDA receptors, we used the model for GluN1/GluN2B NMDA receptor proposed by Amico-Ruvio and Pospescu [37]. This kinetic scheme includes two sequential glutamate binding steps, three pre-open transient states, two desensitization states, and one open state of the receptor (Figure S2A). Because we assume a saturating concentration of glycine inside the clef compartment, the binding steps with this molecule are not included in the kinetic model. Thus, all the resting NMDA receptors are considered glycine bound.

#### 3.1.3. CPC Module

In this module, we implemented a set of equations with the aim of assessing the EPSCs and the respective EPSPs generated by the open fractions of both AMPA and NMDA receptors. EPSPs are then integrated with the back-propagating action potentials programmed during the stimulation design. Finally, the sum of all the depolarizing contributions is used to assess the variations of the post-synaptic membrane potential.

Many synaptic models that have been proposed in the past estimated the EPSCs and/or the EPSPs simply by using two-component exponential functions fitted on electrophysiological recordings [35,58,59,60]. On the contrary, in our model, the open probabilities of the receptors vary according to a system of deterministic rate equations that represent mass-action kinetics of receptors-neurotransmitter interactions [53]. For this reason, the rising and decay phases of both receptor-mediated EPSCs and EPSPs responses are shaped by the complex receptor-specific interaction kinetics with the neurotransmitter. This confers more flexibility to our model, allowing us, for example, to explore the responses generated by mutant forms of the receptors by tuning the rate constants of some of the kinetic equations.

We defined the EPSCs of AMPA and NMDA receptors as follows:

(2) $$EPSC_{AMPA}\left(t\right)=O_{AMPA}\left(t\right)*G_{AMPA}*\left(V_{m}\left(t-\Delta t\right)-V_{E_{AMPA}}\right)$$

(3) $$EPSC_{NMDA}\left(t\right)=O_{NMDA}\left(t\right)*G_{NMDA}*\left(V_{m}\left(t-\Delta t\right)-V_{E_{NMDA}}\right)*B\left(V_{m}\left(t-\Delta t\right)\right)$$

where *O_AMPA_*(*t*) and *O_NMDA_*(*t*) are the number of open NMDA and AMPA receptors at each time step; *G_NMDA_* and *G_AMPA_* are the single channel conductance set to 40 pS and 15 pS, respectively [61,62,63]; *V_m_*(*t* − Δ*t*) is the membrane potential at time (*t* − Δ*t*); *V_E_* is the channel-specific equilibrium reversal potential and defines the value of the membrane potential for which the electrochemical equilibrium is reached and, thus, the net flux through the channel is 0 (we assume that *V_EAMPA_* = *V_ENMDA_* = 0) [64]; and *B*(*V_m_*) describes the voltage dependence of the NMDA current given by the Mg^2+^ blocks defined by [35]:

(4) $$B\left(V_{m}\right)=\frac{1}{1-exp\left(-K_{M}V_{m}\right)*\left(\frac{\left[Mg^{2+}\right]}{3.27}\right)}$$

Once the EPSCs have been calculated, the relative EPSPs are determined simply by applying the law of Ohm:

(5) $${EPSP}_{AMPA}\left(t\right)={EPSC}_{AMPA}\left(t\right){*R}_{s}$$

(6) $$\;EPSP_{NMDA}\left(t\right)=EPSC_{NMDA}\left(t\right)*R_{s}$$

where *R_s_* is the spine’s resistance set to 500 MΩ [65].

Finally, the total membrane potential, defined as the sum of the partial depolarization contributions, is calculated according to the equation:

(7) $$V_{m}\left(t\right)=V_{r}+EPSP_{AMPA}\left(t\right)+EPSP_{NMDA}\left(t\right)+bAP\left(t\right)$$

where *V_r_* is the resting membrane potential of the spine (−65 mV).

In CA3 Schaffer collateral-CA1 synapses, the key mediator of the post-synaptic response is the elicited intracellular Ca^2+^ variation. Because NMDA receptors are the major source of Ca^2+^ during spine stimulation [66], we explicitly calculate the NMDA receptor-mediated Ca^2+^ molar flowrate as follows:

(8) $$\;I_{Ca^{2+}}\left(t\right)=O_{NMDA}\left(t\right)*G_{Ca^{2+}}*\left(V_{M}\left(t-\Delta t\right)-V_{E_{Ca^{2+}}}\right)*B\left(V_{m}\left(t-\Delta t\right)\right)$$

where *G*_*Ca*^2+^_ expresses the permeability of the NMDA receptor to Ca^2+^ ions, set to 2 nM·ms^−1^·mV^−1^ [58] and *V*_*E*_*Ca*^2+^__ is the reversal equilibrium potential for Ca^2+^ set to +130 mV [58].

Finally, the calcium dynamics in the post-synaptic cell is integrated by a simple first-order differential equation [35,58]:

(9) $$\frac{d\left[Ca^{2+}\left(t\right)\right]}{dt}=I_{Ca^{2+}}\left(t\right)-\frac{\left[Ca^{2+}\left(t\right)\right]}{\tau_{Ca^{2+}}}$$

where τ_*Ca*^2+^_ is the passive decay time constant of post-synaptic Ca^2+^ concentration, set to 20 ms [35].

A full list of all the parameters used in the equations described above is provided in the supplementary data (Table S2).

#### 3.1.4. CAS Module

The last module of the pipeline contains a compartmentalized kinetic description of a reaction network that takes place inside the post-synaptic spine. Here, our rationale was to assess the variability in the amount of activated CaMKII enzyme upon different stimulation conditions. Because CaMKII plays a crucial role in the positive regulation of the early phase of LTP in CA3 Schaffer collateral-CA1 synapses [22,23,24], this estimation allows us to qualitatively infer the strength and the efficiency of the synaptic transmission. As previously described for the RGS module, we selected from the literature a kinetic model based on its reproducibility; we translated it inside the PySB framework and, finally, we appended the new block to the pipeline. For this purpose, we selected from the BioModels database [67] a model describing a set of interactions that, starting from post-synaptic rise in Ca^2+^ concentration, leads to the autophosphorylation (i.e., the activation) of monomeric CaMKII [38]. Particularly, the set of reactions implemented includes:

- Binding reactions between Ca^2+^ ions and CaM and CaM-CaMKII species;
- Dimerization reactions between Ca^2+^-CaM and monomeric CaMKII;
- Dimerization reactions between two Ca^2+^-CaM-CaMKII complexes;
- Autophosphorylation reactions of CaMKII monomers inside the 2(Ca^2+^-CaM-CaMKII) complexes.

### 3.2. Data Fitting

#### 3.2.1. Concentration-Response Curves

We computed the glutamate concentration-response curves for NMDA receptors by stimulating the system with a glutamate pulse of 1.5 s in the absence of Mg^2+^ [47]. We run multiple simulations varying the amplitude of the glutamate pulse, with a concentration range between 0.01 and 1500 μM, and calculating the NMDA receptor-mediated current peak values. The EC_50_ values were then calculated by fitting the concentration-response data with the following equation:

(10) $$Response\;\%=\frac{100}{{\left(1+\frac{EC_{50}}{\left[glutamate\right]}\right)}^{n}}$$

where n is the Hill slope.

#### 3.2.2. Two-Component Exponential Function Fitting

The deactivation time constant for the NMDA wild-type receptor and Glu413Gly and Cys461Phe variants were estimated as weighted time constants of the double exponential fit of the NMDA receptor current decay after the exposure of 1 mM glutamate for 1.5 s. The two-component exponential function used for the fitting takes the form:

(11) $$I\left(t\right)=I_{fast}*exp\left(\frac{-t}{\tau_{slow}}\right)+I_{slow}*exp\left(\frac{-t}{\tau_{slow}}\right)$$

where *I* is the current, *I_fast_* and *I_slow_* are the amplitudes of the fast and slow components, respectively, and τ*_fast_* and τ*_slow_* are the respective decay time constants. The weighted time constant of decay (τ*_w_*) was calculated using the following equation:

(12) $$\tau_{w}=\frac{I_{fast}}{I_{fast}+I_{slow}}*\tau_{fast}+\frac{I_{slow}}{I_{slow}+I_{fast}}*\tau_{slow}$$

#### 3.2.3. Four-Parameter Logistic Function and Bending Points

The data generated by the simulation of the relationship between different glutamate-NMDA K_d_ values and the concentration peaks of activated CaMKII enzyme (see results Section 2.4) were fitted with the four-parameter logistic function:

(13) $$Y=\frac{a-d}{1+{\left(\frac{X}{c}\right)}^{b}}+d$$

where *Y* represents the activated CaMKII response, *X* represents the affinity value K_d_ (expressed in μM), *a* is the lower asymptote, *d* is the upper asymptote, *c* represents the K_d_ that generates a mid-way response between the estimated *a* and *d*, and *b* is a slope factor. The bending point of the curve was then computed as follow:

(14) $$X_{bend}=\frac{a-d}{1+k}+d$$

(15) $$Y_{bend}=c*{\left(\frac{a-Y_{bend}}{Y_{bend}-d}\right)}^{\frac{1}{b}}$$

where *k* is a constant value, set to 4.6805 [68].

## 4. Conclusions

We proposed a compartmental model for the hippocampal synapse CA3-CA1. Our goal was to provide a simple and portable, python-based program to run kinetics simulations of the synaptic transmission, which embodied both pre- and post-synaptic activity. The rationale that drove us through the implementation, as well as the application, of this model was to focus on the integration between system biology and structural biology viewpoints. Exploiting this hybrid multiscale approach, we analyzed the impact that single disease associated variants of NMDA receptors, related to neurological disorders and cognitive impairments, may have on the whole synaptic transmission process. We were able to consistently reproduce experimental data and to quantitatively infer molecular-level causality of a variant-related functional impairment. Therefore, these results show the predictive power of such a multiscale approach, aimed at observing behavioral shifts of a complex system that emerge from amplification of small, quantifiable, molecular-level alterations.

A future improvement of our model will allow us to explicitly quantify synaptic plasticity events by adding further biological details, e.g., AMPA receptors conductance modulation and translocation by CaMKII-mediated phosphorylation. The next step will be to extend the structural analysis to the multiple molecular entities involved in the transmission and modulation processes, recalibrating the kinetic constants of the interactions according to the conformational rearrangements caused by specific mutations. This will allow us to explicitly simulate the molecular effects, as well as the impact on the single-neuron functionality, of mutational signatures linked to neurological and cognitive impairments, which affect one or multiple entities of the modeled interactome. Finally, this approach may be extended to post-synaptic receptors belonging to other families, such as G-protein coupled receptors.

## Figures and Tables

**Table 1:** Predicted K_d_ and deactivation time constant for NMDA Wt and variants. Deactivation decay was fitted with a two-component exponential function, and the weighted Tau was then calculated (see methods Section 3.2.2).

	Predicted K_d_ (μM)	Weighted Tau (ms)	
		Predicted	Exp.
Wt	2.5	328	314–570 [47,48,52]
Glu413Gly	190.5	29	20–34 [47,48]
Cys461Phe	446.5	27	28 [48]

## Data Availability

All the code used for implementing and simulating the compartmentalized model is stored in a publicly available github repository (https://github.com/pietromicheli/CA3-CA1_SynapticModel) where a jupyter notebook file for running simulations and performing basic analysis can be also found.

## Supplementary Materials

Supplementary Materials can be found at https://www.mdpi.com/1422-0067/22/4/1536/s1, Figure S1: AMPA-mediated EPSPs generated by pre-synaptic stimulations composed of a single glutamate pulse or a burst of 5 glutamate pulses delivered at 100 Hz, Figure S2: Kinetic schemes used for simulating the gating mechanisms of NMDA and AMPA receptors, Table S1: Comparison between peak open probability and deactivation time constants values obtained from our implementation and the ones reported by the original models, Table S2: List of the parameters used in the equations of the CPC module.

## Abbreviations

AMPA	α-amino-3-hydroxy-5-methyl-4-isoxazolepropionic acid
bAP	back-propagating action potential
CaM	Calmodulin
CaMKII	Ca^2+^/CAM-dependent Kinase II
CAS	CaMKII Activation Simulation
CPC	Current/Potential Calculation
LTD	Long Term Depression
LTP	Long Term Potentiation
mCaMKII	Individual subunits of Calmodulin Kinase II
NMDA	N-Methyl-D-aspartic acid
RGS	Receptors Gating Simulation
STDP	Spike Timing Dependent Plasticity
