# Supplementary material for: A Mechanistic Model of NMDA and AMPA Receptor-Mediated Synaptic Transmission in Individual Hippocampal CA3-CA1 Synapses: A Computational Multiscale Approach
Source: Int J Mol Sci. 2021 Feb 3;22(4):1536. doi: 10.3390/ijms22041536 (PMC7913719; doi:10.3390/ijms22041536)
Supplement: Supplementary file 1 [file ijms-22-01536-s001.pdf]

Supplementary Data

Tab S1 *Comparison between peak open probability and deactivation time constants values obtained from our implementation and the ones reported by the original models. Values were obtained by simulating the respective models with 1 glutamate pulse of 1 mM amplitude and 1 ms of glutamate width.*

	Peak open probability		Deactivation time constant (ms)	
	PySB model	Original model	PySB model	Original model
AMPA	0.32	0.32 [36]	0.54	0.52 [36]
NMDA	0.27	0.27 [37]	162	173 [37]

Tab S2 *List of the parameters used in the equations of the CPC module.*

Parameter	Value	Parameter	Value
V_{max}	+67 (mV)	V_{AMPA}	0 (mV)
I_{fast}	0.75	V_{NMDA}	0 (mV)
I_{slow}	0.25	K_M	0.093 (mV^{-1})
τ_{fast}	3 (ms)	R_S	500 (MΩ)
τ_{slow}	25 (ms)	G_{Ca2+}	0.002 ($\mu M \cdot ms^{-1} \cdot mV^{-1}$)
G_{AMPA}	15 (pS)	V_{Ca2+}	130 (mV)
G_{NMDA}	40 (pS)	τ_{Ca2+}	20 (ms)

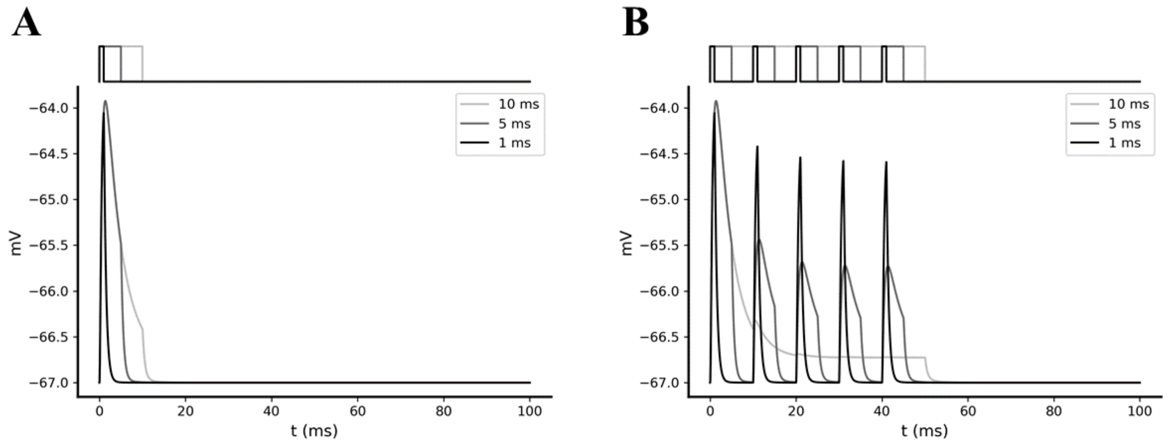

Fig S1 *AMPA-mediated EPSPs generated by pre-synaptic stimulations composed of* ***(A)*** *a single glutamate pulse or* ***(B)*** *a burst of 5 glutamate pulses delivered at 100 Hz. For each stimulation pattern glutamate pulses of 1 mM amplitude were simulated with different widths of 1 ms (black traces), 5 ms (dark gray traces) and 10 ms (light gray traces). We can observe that a small temporal summation only occurs with a pulse duration of 10 ms, reflecting the fast deactivation and desensitization kinetics predicted by the model. Simulations were performed in the presence of 20 AMPA, 15 NMDA and 1 mM* Mg^{2+}.

A

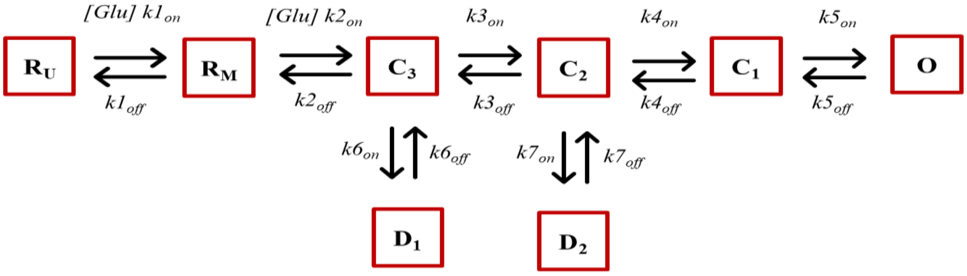

B

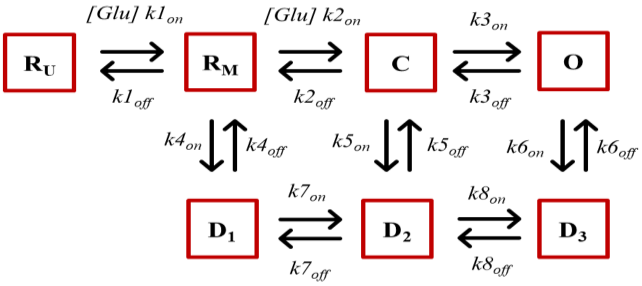

Fig S2 *Kinetic schemes used for simulating the gating mechanisms of* ***(A)*** *NMDA and* ***(B)*** *AMPA receptors.* R_U *represent unbound states,* R_M *represent mono-liganded states (bound to one glutamate),* C_n *represent closed, fully bound states (bound to two glutamate),* D_n *represent desensitized states and O represent open states. All the reaction rate constants can be found in the respective articles [36,37].*
